# Genotyping and distribution of *Giardia intestinalis* assemblages in NSW, Australia

**DOI:** 10.1017/S0031182025100991

**Published:** 2026-01

**Authors:** Patricia Zajaczkowski, Rogan Lee, Damien Stark, Abela Mahimbo, Michael Wehrhahn, Kate Alexander, Stephanie Fletcher-Lartey, John Ellis

**Affiliations:** 1 Faculty of Science, School of Life Sciences, University of Technology Sydney, Ultimo, NSW, Australia; 2 Centre for Infectious Diseases and Microbiology Laboratory Services, ICPMR, Westmead Hospital, Westmead, NSW, Australia; 3 Westmead Clinical School, Faculty of Medicine and Health, The University of Sydney, Westmead Hospital, Westmead, NSW, Australia; 4 Department of Microbiology, St Vincent’s Hospital Sydney, Darlinghurst, NSW, Australia; 5 Faculty of Health, School of Public Health, University of Technology Sydney, Ultimo, NSW, Australia; 6 Microbiology Department, Douglass Hanly Moir Pathology, Macquarie Park, NSW, Australia; 7 Public Health Unit, South-Western Sydney Local Health District, Liverpool, NSW, Australia

**Keywords:** assemblage, diarrhoea, epidemiology, genotyping, *Giardia intestinalis*, giardiasis

## Abstract

Giardiasis is the most common enteric protozoan infection notifiable in New South Wales (NSW), Australia. Surveillance by NSW Health had shown a steady increase (prior to the COVID-19 pandemic) in the number of cases reported since 2012 and the reasons for this currently remain unknown. This study aimed to investigate the occurrence of *Giardia intestinalis* assemblages causing human infection in NSW. Individual faecal specimens were collected from participating hospitals and private laboratories, and the presence of *Giardia* and co-infections was confirmed by real-time multiplex-polymerase chain reaction (PCR). Samples were genotyped by sequence analysis of the triose phosphate isomerase (*tpi*) gene and the small subunit rDNA. Combined genotyping showed that most samples belong to assemblage B, and only a small percentage were infected with only assemblage A. Mixtures of assemblages A and B in individuals were relatively common. Co-infections were observed in ∼ half of the cases, with the most common co-infection being *Blastocystis hominis* and *Dientamoeba fragilis*. Although giardiasis was more prevalent in males, the assemblage distribution between the sexes appeared uniform. The age distribution was bimodal, with peaks in 0–15-year-olds and in adults in their 30s. The overall largest number of cases was collected from patients aged 30–49 years. Interestingly, females aged 5 years old and under had a greater risk of assemblage B infection than their male counterparts. No significant correlation was found between assemblage and clinical symptoms. This study provides new insights into the molecular diversity of giardiasis in NSW and helps inform enhanced surveillance and prevention strategies in Sydney.

## Graphical abstract

**Figure:**
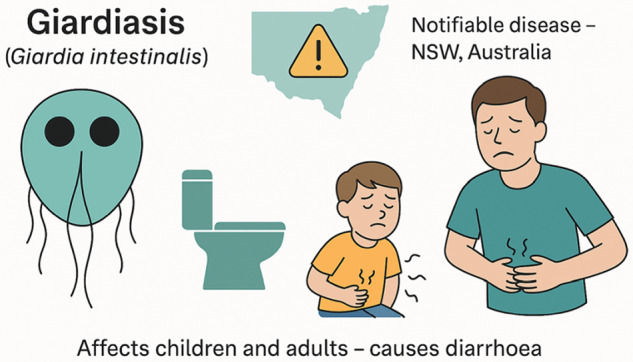

## Introduction

*Giardia intestinalis* continues to be the most encountered parasitic disease worldwide. It is recognized as a significant public health concern and is included in the World Health Organization’s Neglected Diseases Initiative (Savioli et al. 2006). Although more frequently detected in developing countries with limited access to clean water, sanitation and hygiene facilities, ongoing disease surveillance in developed nations has observed a sharp increase in the number of *Giardia* sp.-associated waterborne outbreaks of gastrointestinal illness (Efstratiou et al. 2017; Ryan et al. 2017). Sporadic cases are also on the rise in countries like Australia (Zajaczkowski et al. 2018). In New South Wales (NSW), Australia, giardiasis remains the most common notifiable parasitic infection in NSW with an average (before the COVID-19 pandemic) of around 3000 cases notified by laboratories each year (Communicable Diseases Branch 2017; Zajaczkowski et al. 2018). It may be that these case numbers are reflective of better diagnostic methodologies used in pathology laboratories and the implementation of sensitive assays such as multiplex-PCR. Although it should be noted that culture-independent DNA-based testing methods were only implemented in Australian diagnostic laboratories in late 2013 and onwards, the complete impact of these testing methods on *Giardia* case notifications remains unquantified (Zajaczkowski et al. 2018). The increase of children attending child-care centres across NSW may also play a part in larger case numbers. By state, NSW has the largest share of children attending child-care centres, and use of these centres has increased by 27·8% just within the past 10 years (Department of Education, Employment and Workplace Relations 2022). However, only a few Australian studies have examined the risk factors that drive local transmission of giardiasis (Waldron et al. 2011; Fletcher et al. 2014; Zajaczkowski et al. 2018).

Annual notifications of *G. intestinalis* peak between January and April each year and are highest among children aged 0 to 4 years, and adults aged 30 to 39 years (Communicable Diseases Branch 2017). Incidence rates (per 100 000 population) also vary across communities, ranging from 11·2 to 63·2; and high rates (∼39·0) are reported from both rural and urban health districts (Communicable Diseases Branch 2018). In NSW, it remains unclear if associations exist between high-risk age groups. A recent epidemiological study found that among hospitalized patients in NSW, giardiasis was the second most identified enteric protozoa (after *Blastocystis* spp.) affecting mainly children at school age and younger (Fletcher et al. 2015). Additionally, a more recent study based in South-Western Sydney confirmed that children aged 5-years of age and under were 7 times at greater risk of contracting giardiasis (Zajaczkowski et al. 2018). Despite this, it remains unclear whether there are other factors at play, or even whether fluctuations across NSW communities are reflecting different disease dynamics.

Recent molecular studies have determined that *G. intestinalis* can be split into 8 morphologically identical genetic assemblages (assemblages A to H) which can only be distinguished by molecular typing methods (Feng and Xiao 2011; Adam et al. 2013). Both assemblages A and B are potentially zoonotic; they are responsible for most human infections; however, they have also been successfully isolated from other mammals. The remaining assemblages (C to H) are host-specific, although in some rare cases have been detected in humans (Lasek-Nesselquist et al. 2009; Feng and Xiao 2011). Previous work has shown conflicting results regarding the relationship between *G. intestinalis* assemblages A and B and their clinical presentation. Some studies have suggested that assemblage A is associated with more severe clinical symptoms in Peru, Bangladesh and Spain (Haque et al. 2005; Peréz Cordón et al. 2008; Minetti et al. 2015); however, the opposite has also been suggested by others (Homan and Mank 2001). Currently, studies from Australia have yet to find a clear correlation between assemblages and symptoms in humans despite several genotyping studies being reported (Nolan et al. 2013; Asher et al. 2014, 2016; Ebner et al. 2015; Zahedi et al. 2017). Two studies, however, did investigate a link between clinical symptoms and assemblage type (Read et al. 2002; Yang et al. 2010). Both studies were based in Western Australia. Read et al. (2002) observed a strong association between assemblage A infection and diarrhoea, while Yang et al. (2010) did not find similar correlations. It is difficult to ascertain whether these conflicting results were the result of differences in study methodology; however, it is an issue that needs further investigation.

Clinical symptoms of *G. intestinalis* infection can differ according to each individual, and some cases can even remain asymptomatic. Symptoms can include acute and/or chronic diarrhoea, stomach cramps, nausea, vomiting, dehydration and weight loss (Muhsen and Levine 2012). Although it is still unclear why certain cases remain asymptomatic, it has been suggested that host-parasite factors and the genotypic differences within a parasite can influence the subsequent clinical presentation of an infected individual (Rafiei et al. 2013; Coelho and Singer 2018).

The aim of this study was to identify *G. intestinalis* assemblages contributing to human infections in NSW, Australia, and to detect any significant associations between assemblages and the demographic, clinical and geographical factors. This study provides information on the impact of giardiasis on human health in NSW, and a better understanding of the continuing rise in infection. This will increase the capacity of NSW to apply advanced analyses to disease surveillance and will inform the application of similar methodologies to other intestinal protozoan diseases in NSW.

## Materials and methods

### Faecal specimen collection

Faecal specimens were collected between June 2018 and December 2019 from individuals who had tested positive for *Giardia* species. While the collection process began following ethics approval in 2018, some samples accessed from participating hospitals and private laboratories were retrospectively included, with dates back to 2016. These archived samples were de-identified and their inclusion was permitted under the approved ethics protocol. Samples were collected from 2 hospitals, the Centre for Infectious Diseases and Microbiology at Westmead Hospital, NSW and Sydpath at St. Vincent’s Hospital, NSW. To mitigate geographical bias, samples were also collected from 2 private pathology laboratories, namely Laverty Pathology and Douglass Hanly Moir Pathology (DHM), both situated in NSW, Australia. These private laboratories cover a broader geographical scope within NSW when compared to the hospital laboratories.

Diagnosis of *Giardia* in the hospital pathology laboratories involved a combination of multiplex-PCR detection and immunoassays (Weitzel et al. 2006; Stark et al. 2011; Couturier et al. 2015). In both private pathology laboratories, *Giardia* diagnosis was made by visualizing *Giardia* cysts and/or trophozoites in faecal smears of prepared concentrates using microscopy. Stool samples from DHM were initially prepared using the Mini-Parasep solvent-free (Apacor, England, UK) faecal parasite concentrator with a formalin and Triton X/ethyl acetate solution. Both private laboratories also utilized commercial antigen tests such as the Remel ProSpecT *Giardia*/*Cryptosporidium* microplate immunoassay (Thermo Fisher Scientific) to detect positive antigens.

For each positive stool sample collected, efforts were made to obtain the corresponding patient’s gender, age and postcode region of residence was obtained from the electronic medical records (eMR). To protect the sensitive personal information of the patients, no identifiers were collected from the eMR, and ages of the patients were replaced by age groups to further reduce the possibility of re-identification. All patient ages were categorized into 1 of the 6 age groups: ≤ 5, 6–15, 16–29, 30–49, 50–69 and 70 + years. The history of the patient’s symptoms and potential risk factors were collected from the clinical notes recorded on laboratory requests or medical records. All faecal samples were transported to University of Technology Sydney, provided a unique identification number and stored unpreserved at 4°C before DNA extraction. Note that for individuals with multiple faecal samples collected at the same time, only 1 sample was included in the analyses.

### Multiplex RT-PCR

To confirm the presence of *G. intestinalis* and to detect any co-infections within the collected samples, the specimens were analysed by a multiplexed real-time PCR (RT-PCR) EasyScreen assay (Genetic Signatures, Newtown, Australia) at Sydpath at St. Vincent’s Hospital, Sydney, Australia (Stark et al. 2011). The assay includes a DNA extraction step as part of the workflow, using proprietary 3base technology to enhance detection sensitivity. The EasyScreen kit tests for a variety of enteric pathogens including common enteric protozoan parasites: (a) *Dientamoeba fragilis*, (b) *Cryptosporidium* spp., (c) *Blastocystis hominis*, (d) *Entamoeba* complex, (e) *G. intestinalis*; bacterial pathogens: (a) *Salmonella* spp., (b) *Campylobacter* spp., (c) *Shigella* spp., (d) *Yersinia enterocolitica*, (e) toxigenic *Clostridium difficile* and (f) *Listeria monocytogenes*; and viruses: (a) Norovirus group I, (b) Norovirus group II, (c) Adenovirus hexon, (d) Adenovirus 40/41, (e) Rotavirus A and B, (f) Astrovirus (group 1–7) and (g) Sapovirus.

### Stratified sample selection

During the study period, a total of 410 *G. intestinalis*-positive faecal samples were collected. Of these, 107 were excluded due to missing or incomplete demographic and clinical data (i.e. more than half the fields unavailable), leaving 303 samples. From this group, 169 (55*·*8%) were selected for genotyping using a stratified sampling strategy to ensure proportional representation by age, sex and geographic location.

While efforts were made to obtain full demographic data for all 169 selected samples, a small proportion (4*·*1%) had 1 or 2 missing variables (e.g. gender, age, postcode, seasonality, travel history or risk factors). These samples were still included, as they provided valuable insight into assemblage distribution across NSW. This limitation is acknowledged, and readers are advised that conclusions drawn from the demographic trends should be interpreted with caution due to the small number of incomplete records.

Sample collection occurred continuously over the 42-month study period, and samples underwent DNA extraction and genotyping in sequential batches rather than in a single selection at the end of the study. To account for temporal variation, stratified sampling was applied within defined 3-month intervals, aligning with seasonal time periods. Within each interval, samples were selected to proportionally represent the demographic and geographic characteristics of the total case population during that timeframe.

All faecal specimens were stored unpreserved at 4°C before DNA extraction. The final genotyped cohort of 169 samples was constructed by aggregating each stratified batch, ensuring the resulting dataset reflected the demographic, geographic and temporal composition of the full sample population. This methodology helped minimize potential bias related to sample reduction.

### Genomic DNA extraction

To prepare samples for genotyping, a DNA extraction was performed on 150 mg of each of the 169 collected faecal samples using the ISOLATE II Fecal DNA Kit (Bioline, Sydney, Australia) according to the manufacturer’s instructions with minor modifications. Specifically, the wash step was performed 3 times with Fecal DNA Wash Buffer (rather than once as per the manufacturer’s instructions). Elution was accomplished by adding 100 μl elution buffer. The eluted DNA was stored at −20°C until PCR amplification. Samples with sterile water were used as a negative control to monitor contamination during nucleic acid extraction. Samples spiked with *Cryptosporidium* spp. DNA templates were used as a positive control during the extraction process. DNA concentration and purity were determined via 260/280 and 260/230 ratios measured on the NanoDrop One microvolume UV-Vis Spectrophotometer (Thermo Fisher Scientific, United States).

## Nested PCR amplification of the G. intestinalis SSU rRNA

Primers originally designed by Hopkins et al. (1997) amplify the small subunit ribosomal RNA (SSU-rRNA) gene of *G. intestinalis* producing a 292 bp product from the primary PCR reaction, and a 130 bp product from the secondary PCR reaction (Hopkins et al. 1997). Due to the small product size and innate low genetic variation within the SSU-rRNA gene, distinguishing between assemblage A and B sequences is entirely dependent on identifying 4 single nucleotide polymorphisms (SNPs). To increase discriminatory power, new primers were designed to capture an area with a greater distribution of polymorphisms between assemblages A and B. The Clustal W multiple sequence alignment program was used to align SSU-rDNA sequence reads of assemblages A and B, and a total of 7 SNPs were identified between the 2 assemblages (Larkin et al. 2007).

A 447 bp fragment of the SSU-rRNA gene was first amplified using the previously described forward primer RH11 (Hopkins et al. 1997) and the newly designed reverse primer RH4.1: TGGCACCAGACCTTGCCCT. This reaction was followed by a secondary amplification step, which used the internal primer GiarF (Read et al. 2002) and the newly designed reverse primer GiarR.1: ACTCCCCGTCGCTGCCT. The use of GiarR.1 resulted in a nested PCR amplicon of 363 bp long. Both PCR amplifications were prepared in a final volume of 50 μl and carried out using conditions previously described (Hopkins et al. 1997). Negative controls (no template added) and positive controls (containing DNA from previously sequenced and confirmed *G. intestinalis* samples) were included in each assay reaction. Reactions were performed on an Eppendorf Mastercycler Nexus (Sigma-Aldrich).

Specificity of the novel primers was tested using a panel of 3 protozoan parasite-positive and 2 bacteria-positive clinical samples previously submitted to St Vincent’s Hospital (including *Cryptosporidium parvum, D. fragilis, B. hominis, Campylobacter* spp. and *Clostridium* spp.). Sensitivity was estimated using a series of 10-fold dilutions of DNA from extracted *G. intestinalis* DNA samples to assess the lowest detection threshold of each PCR assay. Reaction templates corresponded to decreasing concentrations from 10^2^ to 10^−3^ ng/µL DNA per PCR tube.

To confirm successful amplification, 4 µL of the PCR product was subjected to electrophoresis on a 2·0% agarose gel containing GelRed Nucleic Acid Gel Stain (Sigma-Aldrich). PCR products of the correct band length (363 bp) were purified by using a PCR purification kit (Qiagen, GmbH. Germany) and sequenced (Macrogen, Seoul, Korea) on both strands using the PCR primers. Sequence data were trimmed and analysed using SeqTrace and for each PCR product, a consensus contig was generated from the sequence data (Stucky 2012). The final sequences were then compared to sequences (>99% similarity) contained in GenBank using the nucleotide-BLAST tool. The identification of homologous sequences allowed the determination of the *G. intestinalis* assemblage. Mixed-assemblage infections were identified by the presence of clear and consistent double peaks at all 7 SNP positions in the chromatograms, indicating the simultaneous presence of both Assemblage A and B templates within the same sample.

### Assemblage-specific nested PCR amplification of the TPI gene

Two *G. intestinalis* assemblage-specific nested PCR assays were used to amplify the triose phosphate isomerase (TPI) gene. A 605 bp fragment of the gene was first amplified using previously described primers AL3543 and AL3546 (Sulaiman et al. 2003). The secondary PCR reaction involved 2 separate assays using assemblage-specific primers; the assemblage A-specific primers Af and Ar amplifying a 332 bp PCR product (Geurden et al. 2008) and the assemblage B-specific primers Bf and Br amplifying a 400 bp product (Levecke et al. 2009). Both PCR amplifications were prepared in a final volume of 50 μl and carried out using conditions previously described (Levecke et al. 2009). Negative controls (no template added) and positive controls (containing DNA from previously sequenced and confirmed *G. intestinalis* samples) were included in each assay reaction. Reactions were performed on an Eppendorf Mastercycler Nexus (Sigma-Aldrich) and PCR products were separated by electrophoresis in a 2·0% agarose gel containing GelRed Nucleic Acid Gel Stain (Sigma-Aldrich).

### Defining single and mixed assemblage infections

Further genetic characterization of *G. intestinalis* involved combining the results of both the assemblage-specific PCR (*tpi*) and the nested PCR (SSU-rRNA). Characterizing a sample as a single assemblage (either assemblage A or B) would require both PCR assays to have an identical result. A mixed assemblage was defined by either an identical A + B result for both PCR assays or discordant results from each assay.

### Statistical analyses and mapping of spatial data

Statistical analysis was performed using SPSS Statistics 27 (IBM, USA). Categorical variables are reported in terms of percentages, with corresponding confidence intervals (CI) at 95%. The existence of association between categorical variables was evaluated using Pearson’s Chi-Square test (or Fisher’s Exact test for sparse data). Statistical significance was set as a *P*-value < 0·05.

Positive human cases of giardiasis in NSW were also geographically mapped using ArcGIS. Case postcode data was initially geocoded using ArcGIS, then spatially joined to 2 polygon layers: NSW Local Government Area (LGA) boundaries (2019) and Local Health District (LHD) boundaries (2014). NSW is divided into 8 metropolitan LHDs and 7 rural/regional LHDs34. The LHDs are further split into 128 LGAs. Cases were then aggregated according to the postcode-matched LGA and LHD, to calculate the total number of *G. intestinalis* cases and *G. intestinalis* assemblages for each region in NSW.

## Results

### *Detection and identification of* G. intestinalis *genotypes*

Of the 169 samples selected through stratified sampling and subjected to genotyping, the majority (87·0%) were sourced from private pathology laboratories, while a smaller proportion (13·0%) originated from hospital laboratories. The *tpi* assemblage A/B-specific PCR amplified in 87·0% (147/169) of these samples; 18·4% (*n* = 27) were only assemblage A, 54·4% (*n* = 80) were only assemblage B and 27·2% (*n* = 40) were classified as mixed assemblages. The SSU-rRNA PCR amplified 80·5% (136/169) of the specimens; of which 18·4% (*n* = 25) were only assemblage A, 73·5% (*n* = 100) were only assemblage B and 8·1% (*n* = 11) were mixed assemblages A + B. An overview of these sample numbers and distributions is provided in Supplementary Table 1.

Among the subset of *G. intestinalis*-positive samples that were collected, 162 (95·9%) were successfully amplified at 1 or more loci; of which 9·3% (*n* = 15) were only assemblage A, 46·9% (*n* = 76) were only assemblage B and 43·8% (*n* = 71) were mixed infections of assemblage A + B (see Supplementary Table 1).

Co-infecting pathogens were detected in 49·1% (*n* = 83) of all *G. intestinalis*-positive faecal samples. Of these co-infections, 56·6% (*n* = 47) were parasitic, 12·0% (*n* = 10) were bacterial and 8·4% (*n* = 7) were viral. Joint parasitic/viral co-infections were also identified in 14·5% (*n* = 12), followed by parasitic/bacterial co-infections (4·8%, *n* = 4), bacterial/viral co-infections (2·4%, *n* = 2) and parasitic/bacterial/viral co-infections (1·2%, *n* = 1). Overall, the most common pathogens detected were the enteric protozoa *B. hominis* (31·3%, *n* = 25) and *D. fragilis* (15·0%, *n* = 12), followed by the pathogens *Campylobacter* spp. (5·0%, *n* = 4) and *Enterovirus* (5·0%, *n* = 4). Other co-infections are reported in Supplementary Table 2.

### *Socio-demographics of* G. intestinalis *assemblage cases*

**Figure 1.**
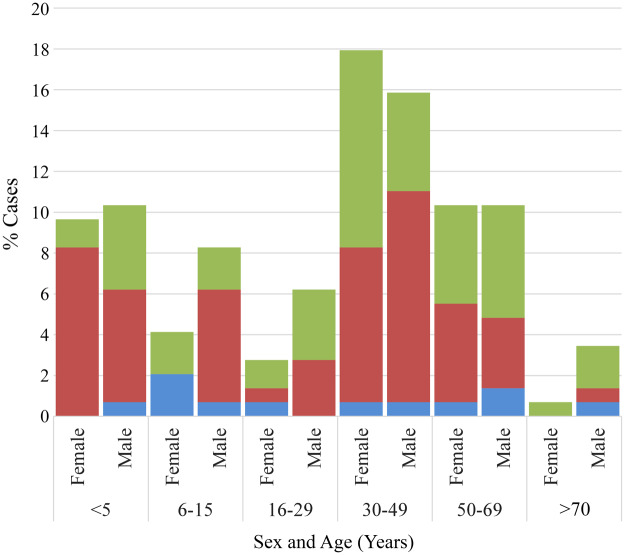
Distribution of *G. intestinalis* assemblages A, B and A + B by age and sex (%). Assemblage A, blue; assemblage B, red; mixed-assemblage A + B, green.

Of the 169 *G. intestinalis* positive cases selected to represent the broader population, 85·8% (145/169) had complete age metadata and 91·1% (154/169) had valid gender metadata; these cases were included in subsequent analyses (see Supplementary Table 1). Combined age and gender distributions are presented in Figure 1. Individuals infected with *G. intestinalis* ranged in age from infancy to over 70 years. A bimodal age distribution was observed, with peaks among children aged ≤ 5 years and adults in their 30s. Cases aged 30–49 (33·8%, *n* = 49) and 50–69 years (20·7%, *n* = 30) made up the largest age groups, followed closely by those ≤ 5 years (20·0%, *n* = 29).

Overall, giardiasis was observed in more males (55·2%, *n* = 85) than females (44·8%, *n* = 69), although there was no significant difference between these 2 groups (Table 1). Within male and female groups, the assemblage distribution appeared uniform. For males, single infections with assemblages A or B were identified in 53·8 and 57·5% cases, respectively. Among females, 46·2% cases were assemblage A only infections, whilst 42·5% cases were only B. Mixed assemblage A + B cases were identified in 52·9% males and 47·1% females. Assemblages were distributed across all age groups; however, single assemblage A infections were mostly seen in children aged 6–15 years, and adults aged 50 years and greater. In comparison, single assemblage B infections were more common among middle-aged individuals aged 30–49 years old, and children aged 5 years and under. When categorizing the cases into 2 age categories (≤5 and > 5 years), it was found that children ≤ 5 years old were more commonly infected by assemblage B only (OR = 2·74; 95% CI: 1·15–6·51; *P* = 0·020) than assemblage A only (Table 1). Additionally, females aged ≤ 5 years old had a greater risk of assemblage B-only infection than their male counterparts (OR = 2·61; 95% CI: 1·12–6·07; *P* = 0.001).

**Table 1.** Distribution of *G. intestinalis* assemblages based on age (*n* = 145) and sex (*n* = 154)

	Assemblage A			Assemblage B			Assemblage A + B			
Demographics	% (*n*)	*P*-value	OR (95% CI)	% (*n*)	*P*-value	OR (95% CI)	% (*n*)	*P*-value	OR (95% CI)	Total, % (*n*)
*Sex*										
*Male*	53·8% (7)	0·919	0·942 (0·30–2·95)	57·5% (42)	0·579	1·197 (0·63–2·26)	52·9% (36)	0·617	0·85 (0·45–1·61)	55·2% (85)
*Female*	46·2% (6)			42·5% (31)			47·1% (32)			44·8% (69)
*Age (years)*										
*≤5*	8·3% (1)	0·275	–	27·8% (20)	0·104	–	13·1% (8)	0·299	–	20·0% (29)
*6–15*	33·3% (4)			11·1% (8)			9·8% (6)			12·4% (18)
*16–29*	8·3% (1)			6·9% (5)			11·5% (7)			9·0% (13)
*30–49*	17·0% (2)			36·1% (26)			34·4% (21)			33·8% (49)
*50–69*	25·0% (3)			16·7% (12)			24·6% (15)			20·7% (30)
*70 +*	8·3% (1)			1·4% (1)			6·6% (4)			4·1% (6)
*Age (years – 2 categories)*										
*≤5*	8·3% (1)	0·263	0·341 (0·04*–*2·75)	27·8% (20)	0·020*	2·74 (1·15–6·51)	13·1% (8)	0·077	0·45 (0·19–1·11)	20·0% (29)
*>5*	91·7% (11)			72·2% (52)			86·9% (53)			80·0% (116)

* *P*-value < 0·05 is significant.

### *Clinical and travel history of* G. intestinalis *cases*

Overall, no significant association was found between symptoms and infection with specific *G. intestinalis* assemblages. Of the 9 asymptomatic cases, 5 had mixed assemblages A + B, 3 had assemblage B and 1 had assemblage A. Common clinical symptoms identified from the *G. intestinalis* cases included diarrhoea (79 0%, *n* = 49), abdominal pain (21 0%, *n* = 13) and bloating (19 4%, *n* = 12) (Table 2). Vomiting and/or nausea, weight loss and fatigue were also reported, albeit rarely (8 1, 1 6 and 1 6%, respectively). A few individuals positive for *G. intestinalis* also reported being asymptomatic (14 5% (*n* = 9)) at the time of sampling. These included cases who had recently travelled overseas and/or arrived in the country as a refugee (*n* = 3), as well as family members, friends and household members of confirmed giardiasis cases (*n* = 5). The remaining asymptomatic case (1 6%) had reported having a lowered immunity. A total of 62 cases whose isolates were successfully genotyped (and who did not have co-infections with other enteropathogens) had clinical data available (Table 2). More cases from whom assemblage B and mixed assemblages A + B were identified reported symptoms (*n* = 32 and *n* = 27, respectively); however, comparisons could not be made with assemblage A as clinical data was only available for 3 individuals. Regarding the travel history of cases, 7 8% (*n* = 10/129) of cases reported travelling overseas prior to illness onset. Out of the 10 cases, none were infected with assemblage A only, 20 0% had assemblage B and the remaining 80 0% were found to have mixed A + B infection. Additionally, those who had travelled overseas were 6 times more likely to be infected with mixed assemblages A + B (OR = 5 917; 95% CI: 1 20–29.08, *P* = 0.02) as opposed to single infections.

**Table 2.** *G. intestinalis* assemblages and recorded clinical symptoms

	Assemblage A (*n* = 3)			Assemblage B (*n* = 32)			Assemblage A + B (*n* = 27)			
Clinical data^a^	% (*n*)	*P*-value	OR (95% CI)	% (*n*)	*P*-value	OR (95% CI)	% (*n*)	*P*-value	OR (95% CI)	Total, % (*n*)
*Asymptomatic*	33·3% (1)	0·381	3·188 (0·258–39·360)	9·4% (3)	0·205	0·414 (0·093–1·832)	18·5% (5)	0·334	1·761 (0·424–7·315)	14·5% (9)
*Diarrhoea*	33·3% (1)	0·109	0·115 (0·010–1·380)	84·4% (27)	0·286	1· 964 (0·562–6·862)	77·8% (21)	0·831	0·875 (0·256–2·989)	79·0% (49)
*Bloating*	0·0% (0)	0·518	–	12·5% (4)	0·158	0·393 (0·105–1·476)	29·6% (8)	0·072	3·263 (0·864–12·328)	19·4% (12)
*Abdominal pain*	33·3% (1)	0·513	1·958 (0·164–23·449	15·6% (5)	0·286	0·509 (0·146–1·780)	25·9% (7)	0·400	1·692 (0·494–5·789)	21·0% (13)
*Vomiting and/or nausea*	0·0% (0)	0·774	–	6·3% (2)	0·469	0·600 (0·093–3·867)	11·1% (3)	0·376	2·063 (0·320–13·313)	8·1% (5)
*Weight loss*	0·0% (0)	0·952	–	0·0% (0)	0·484	–	3·7% (1)	0·435	–	1·6% (1)
*Fatigue*	0·0% (0)	0·952	–	3·1% (1)	0·516	–	0·0% (0)	0·565	–	1·6% (1)
*Immunocompromised*	0·0% (0)	0·952	–	0·0% (0)	0·484	–	3·7% (1)	0·435	–	1·6% (1)
*Other symptoms*	0·0% (0)	0·733	–	12·5% (4)	0·367	2·000 (0·339–11·817)	7·4% (2)	0·468	0·620 (0·105–3·666)	9·7% (6)

a Excluding clinical data for samples with co-infections with other enteropathogens.

### *Spatial and seasonal distribution of* G. intestinalis *assemblages across NSW LHDs*

**Figure 2.**
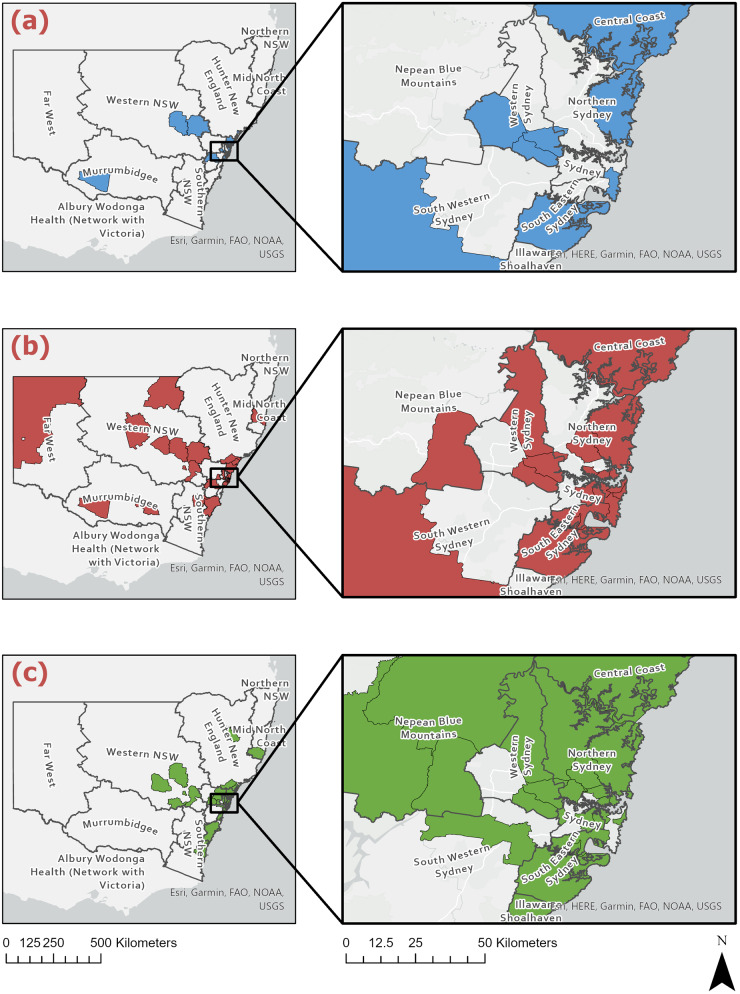
Geospatial distribution of *G. intestinalis* assemblages A, B and A + B across NSW local health districts (*n* = 141). This figure shows the geospatial distribution of (a) *G. intestinalis* assemblage A (blue), (b) *G. intestinalis* assemblage B (red) and (c) *G. intestinalis* mixed-assemblage A + B (green) across NSW local health districts.

Of the 169 giardiasis cases selected to reflect the broader population, only 141 had valid geographic information available and were therefore included in the spatial mapping across NSW (Figure 2), aggregated into assemblage groups. Cases appeared in metropolitan areas of Sydney, the Blue Mountains (west of Sydney) and regional inland and coastal centres of NSW. Most cases, however, were from metropolitan areas, in particular the Northern Sydney district. The surrounding areas of Inner-West Sydney also showed a high frequency of giardiasis infection (Figure 2). In the regional/rural areas, cases were often seen in the Newcastle and lower Hunter region as well as mid-western regional locations including Dubbo, Orange and Bathurst. Allocation of postcode data to LHDs supported this: a total of 67 4% (*n* = 95) samples were from metropolitan LHDs (see Table 3). The Northern Sydney LHD accounted for over a quarter (29 5%, *n* = 28) of all metropolitan samples, and of these 35 7% (*n* = 10) were from the Northern Beaches LGA. A total of 32 6% (*n* = 46) of cases were from rural/regional LHDs, including Western NSW (41 3%, *n* = 19), Hunter New England (26 1%, *n* = 12) and the Far West (10 9%, *n* = 5). A smaller number of regional cases were from Southern NSW (*n* = 4), Murrumbidgee (*n* = 3) and Mid North Coast (*n* = 3).

**Table 3.** Distribution of *G. intestinalis* assemblages based on region of residence in NSW (*n* = 141)

	Assemblage A			Assemblage B			Assemblage A + B			
Local Health District (LHD) of residence	% (*n*)	*P*-value	OR (95% CI)	% (*n*)	*P*-value	OR (95% CI)	% (*n*)	*P*-value	OR (95% CI)	Total, % (*n*)
*Central Coast*	7·7% (1)	0·568	–	12·7% (9)	0·833	–	5·0% (3)	0·350	–	9·0% (13)
*Hunter New England*	7·7% (1)			7·0% (5)			10·0% (6)			8·3% (12)
*Nepean Blue Mountains*	0·0% (0)			4·2% (3)			5·0% (3)			4·2% (6)
*Northern Sydney*	7·7% (1)			16·9% (12)			25·0% (15)			19·4% (28)
*South-Eastern Sydney*	15·4% (2)			11·3% (8)			10·0% (6)			11·1% (16)
*South-Western Sydney*	7·7% (1)			2·8% (2)			1·7% (1)			2·8% (4)
*Sydney*	0·0% (0)			9·9% (7)			5·0% (3)			6·9% (10)
*Western Sydney*	23·1% (3)			8·5% (6)			6·7% (4)			9·0% (13)
*Far West*	0·0% (0)			5·7% (4)			1·7% (1)			5·6% (5)
*Illawarra Shoalhaven*	0·0% (0)			1·4% (1)			6·7% (4)			3·5% (5)
*Mid-North Coast*	0·0% (0)			1·4% (1)			3·3% (2)			2·1% (3)
*Murrumbidgee*	7·7% (1)			2·8% (2)			0·0% (0)			2·1% (3)
*Southern NSW*	0·0% (0)			2·8% (2)			3·3% (2)			2·8% (4)
*Western NSW*	15·4% (2)			11·3% (8)			15·0% (9)			13·2% (19)
*Residence (2 categories)*										
Metropolitan	66·7% (8)	0·597	1·06 (0·31–3·65)	68·6% (48)	0·808	1·091 (0·54–2·20)	66·1% (39)	0·763	0·897 (0·44–1·82)	67·4% (95)
Regional/rural	33·3% (4)			31·4% (22)			33·9% (20)			32·6% (46)

**Figure 3.**
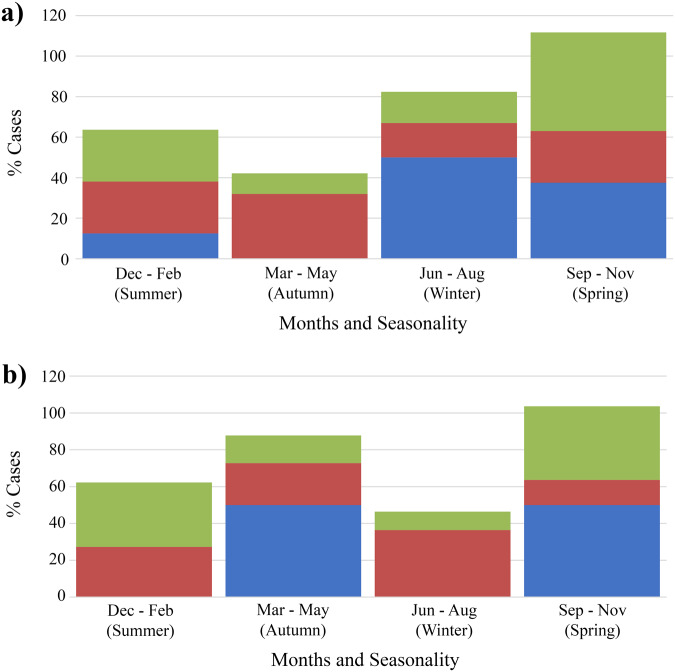
Distributions of *G. intestinalis* assemblages and seasonal dispersal across metropolitan and rural/ regional local health districts. This figure shows the seasonal distribution of genotyped cases occurring in (a) metropolitan local health districts (*n* = 94) and (b) regional/ rural local health districts (*n* = 46) in NSW between 2016 and 2019. Assemblage A, blue; assemblage B, red; mixed-assemblage A + B, green.

Using ArcGIS, the *G. intestinalis* assemblages (*n* = 141) were mapped to LGA boundaries. The maps (Figure 2) showed that the assemblages were distributed across the entirety of NSW and did not show obvious geographic clustering. There were no correlations found between region of residence and specific assemblage type, and most infections were commonly reported in metropolitan regions of Sydney and the eastern coast of NSW. There were, however, significant associations found between seasons and assemblage B only infections (*P* = 0·004) as well as mixed assemblage infections (*P* = 0·005). In metropolitan NSW, single assemblage A infections were not observed in autumn, and were only detected in summer, spring and winter (*P* = 0·048) (Figure 3). Additionally, mixed assemblage infections were most often found in spring (*P* = 0·004), whilst single assemblage B infections made up most cases in summer, autumn and winter. In rural/regional areas of NSW (Figure 3), single assemblage B infections made up 31·0% of all cases, and peaked in autumn and winter.

## Discussion

The present study aimed to identify the genetic diversity of *G. intestinalis* in humans across metropolitan and rural/regional areas of NSW, Australia during 2016–2019. Faecal samples that were positive for *G. intestinalis* were collected (*n* = 410) from participating hospital and private pathology laboratories participating in the study. From this pool, a representative subset of samples was chosen for genotyping (*n* = 169) and 95 9% (*n* = 162) were successfully amplified using PCR. The genotyping results indicate the presence of both assemblages A and B in NSW. Assemblage B infections were predominant, accounting for 46 9%, followed by mixed infections of assemblages A and B at 43 8% and single assemblage A infections at 9 3%. There is still a lot of controversy surrounding the distribution of assemblages around the world mainly because the results are difficult to compare. Studies in Australia, Austria and Sweden determined that assemblage B was the most dominant (Read et al. 2002; Lee et al. 2006; Lebbad et al. 2011). Assemblage B infections are not only more virulent than assemblage A, but have been reported more commonly in giardiasis outbreaks (Xiao and Feng 2017). The higher virulence of assemblage B infections suggests that those infected are more likely to seek medical assistance, which in turn could account for the higher detection rates. High re-infection rates have also been attributed to assemblage B infections, wherein poor hygiene and environmental contamination allow this assemblage to recirculate within a community (Thompson 2008).

Of note is the high number of mixed-assemblage infections identified (43 8%). In other studies, mixed-assemblage infections are rarely identified accurately, and most studies report only a 3–10% prevalence (Kohli et al. 2008). PCR-based studies that use assemblage-specific primers targeting the *tpi* locus are often more likely to observe mixed-assemblage infections in comparison to other PCR methodologies (Huey et al. 2013; Elhadad et al. 2021). In part, this is due to the polymorphic nature of the *tpi* markers that allow them to reliably distinguish between assemblage A and B isolates. While standard primer sets may overlook mixed assemblage cases due to the variable proportions of assemblage A and B DNA, assemblage-specific primers excel in this regard (Zajaczkowski et al. 2021). This was confirmed in the present study, where the *tpi*-PCR successfully genotyped 27 2% mixed assemblage A + B infections, in comparison to the 8 1% obtained by the SSU-rRNA-PCR assay.

In the present study, cases of *G. intestinalis* infection were found across all age groups ranging from 0 years to over 70 years. Data show that the number of cases peaked at ages ≤ 5 years and 30–49 years regardless of gender (Figure 1). Similar bimodal age distributions have been observed in *G. intestinalis* surveillance reports from the USA (Yoder et al. 2012; Painter et al. 2015) and England (Breathnach et al. 2010). Although the distribution of *G. intestinalis* assemblages was found across all age groups in NSW, adults aged in their 30s and 40s, and children under 5 years of age maintained a higher prevalence of assemblage B. In fact, children under 5 years old were more commonly infected by assemblage B (OR = 2 74; 95% CI: 1 15–6 51; *P* = 0 020) than assemblage A. This strong association between assemblage B infection and children under 5 years might be indicative of age-specific risk factors and transmission routes. The exposure to assemblage B organisms in children can occur either through high-risk activities such as day-care attendance or schooling, as well as poor hygiene behaviours (Thompson 2000; Oliveira-Arbex et al. 2016). Children with assemblage B infections have also demonstrated a higher level of cyst shedding, which would facilitate a faster spread within institutional settings and areas where children frequent (Kohli et al. 2008). In Spain, *G. intestinalis*-positive children were 10 times more likely to be infected with assemblage B in comparison to adults (Wang et al. 2019). Additionally, children may play a critical role in ongoing transmission cycles by facilitating secondary spread to family members, day-care centre staff and other children. Nappy changing and toilet training are particularly high-risk activities for parents and caregivers, due to frequent contact with faecal material and possible environmental contamination. The burden of exposure from infected children likely contributes to the increased rates of assemblage B infection observed in parent-aged adults. Supporting this, a Brazilian study observed a predominance of assemblage B infection in middle-aged adults aged 30 to 39 years old (Faria et al. 2016), a pattern also reported in England (Minetti et al. 2015). Co-infecting pathogens were detected in nearly half (49 1%, *n* = 83) of all-*G. intestinalis*-positive faecal samples. Infection of *G. intestinalis* with concomitant infections with a variety of gut bacteria, viruses and parasites are incredibly common in most countries. Most co-infections were parasitic (56 6%, *n* = 47) and bacterial (12 0%, *n* = 10). Overall, the most common pathogens detected were the enteric protozoa *B. hominis* (31 3%, *n* = 25) and *D. fragilis* (15 0%, *n* = 12), followed by *Campylobacter* spp. (5 0%, *n* = 4) and *Enterovirus* (5 0%, *n* = 4). Infection of *G. intestinalis* with concomitant infections with a variety of gut bacteria, viruses and parasites are incredibly common in most countries. In India, *Giardia* infections with *Vibrio cholerae* and rotavirus were commonly identified in children aged under 10 years old (Mukherjee 2014) while in Nicaragua, the majority of *G. intestinalis* cases (70 4%) were co-infected with either *Norovirus, Sapovirus* or enteropathogenic *Escherichia coli* (EPEC). (Becker-Dreps et al. 2014) In the USA, multiple parasites including *G. intestinalis, Cryptosporidium* spp., and *Entamoeba* spp. were responsible for drinking water outbreaks (Craun et al. 2010). Interestingly, a study in Uganda observed a link between *Giardia* assemblage B and *Helicobacter pylori* infection (Ankarklev et al. 2012). There are limited studies on the associations between *G. intestinalis* assemblages and co-infecting pathogens, so comparisons remain difficult to make. In the present study, no associations were found between assemblage type and co-infecting pathogen. The high level of co-infections can be explained as most enteric pathogens are transmitted via the same route of infection: the faecal-oral route. This underscores the value of continued examination of faecal specimen from symptomatic persons for multiple pathogens in developed settings such as Australia, where the practice appears to be diminishing in clinical settings.

In the present study, it was found that clinical symptoms were not associated with assemblage type. This was consistent with previous studies in Brazil (Kohli et al. 2008), Iran (Bahrami et al. 2017; Kashinahanji et al. 2019; Rafiei et al. 2020), Thailand (Tungtrongchitr et al. 2010) and China (Liu et al. 2012). Despite this, there have been other studies that have reported a close association between assemblages and clinical symptoms (Helmy et al. 2009; Al-Mohammed 2011; Pestehchian et al. 2012). Assemblage B has been associated with severe diarrhoea, vomiting, abdominal pain and bloating (Al-Mohammed 2011; ElBakri et al. 2014; Hussein et al. 2017; Wang et al. 2019), although it is equally plausible that since younger children and their middle-aged parents appear to be predominantly affected with this assemblage and are more likely to seek care, these assemblages become overrepresented among notified cases. In other studies, assemblage A has been affiliated with more serious clinical symptoms (Read et al. 2002; Haque et al. 2005; Breathnach et al. 2010; Sarkari et al. 2012; El Basha et al. 2016). It remains difficult to determine a true correlation between assemblages and symptoms. It may be that the virulence of assemblages A and B in humans relies on a variety of factors, including human host age and gender, parasite growth rates, metabolic products or toxins and even drug resistance.

Another interesting finding was that only 8 0% of *G. intestinalis* positive cases reported travelling overseas prior to illness onset, suggesting that most giardiasis cases in NSW are a result of endemic transmission. There is a misconception that *G. intestinalis* infection in industrialized countries is mainly associated with international travel to developing nations. Several studies have observed that most giardiasis cases in industrialized countries are in fact a result of endemic transmission and local risk factors (Espelage et al. 2010; Plutzer et al. 2010; Woschke et al. 2021). In the present study, individuals with a history of overseas travel were 6 times more likely to be infected with mixed assemblages A and B (OR = 5 917; 95% CI: 1 20–29 08, *P* = 0 02) as opposed to being infected with a single assemblage infection. This is a novel finding which has not been observed elsewhere, and it remains important to investigate this further. However, a recent study (Samie et al. 2020) noted that the occurrence of mixed-assemblage infections is higher in developing countries as opposed to developed regions of the world. Exposure to conditions where environments are contaminated with human faeces, have poorer access to or less well-maintained hygiene facilities may be more common in developing settings, which increases the risk of both assemblages co-circulating in communities and being picked up by travellers.

Analyses of 141 cases of sporadic human giardiasis showed that infections were widely dispersed across eastern regions of Sydney and NSW, where the majority of the NSW population resides (Australian Bureau of Statistics 2017) (Figure 2). Among the NSW LHDs, most (67 4%) sporadic cases occurred in metropolitan LHDs. These findings are consistent with historical state-wide surveillance trends that identified significant positive associations between area-level advantage and an increased likelihood of giardiasis notifications (Mazumdar et al. 2020). However, it cannot be ignored that the high incidence rates of giardiasis detected in urban Sydney may also be artefactual, particularly as these locations often have highly transient populations. It must also be considered that individuals residing in metropolitan areas have better access to primary healthcare facilities and greater access or inclination to submitting stool samples for testing when compared with those living in rural areas. In addition to this, densely populated cities such as Sydney have a higher risk of exposure to an infected individual, whether that be through contaminated environment, wastewater, sewage or recreational waters or transmission through day-care centres, schools, and other institutional settings. The plausibility of this was confirmed by a study in the USA that found a positive correlation between giardiasis prevalence and population density and population size (Dreelin et al. 2014).

Seasonal trends in the dispersal patterns of assemblages A and B were also observed (Figure 3.a and Figure 3.b). Single assemblage A cases were not detected in metropolitan LHDs during autumn (*P* = 0 048). Alternatively, single assemblage A cases were also missing in regional areas across summer and winter. This finding may be artefactual and is likely due to the lower numbers of assemblage A cases identified throughout the study. The low number of single Assemblage A infections may also reflect methodological limitations, particularly the reduced sensitivity of some assays to detect minor assemblage components in mixed infections. Rather than indicating a true scarcity of Assemblage A, it is possible that these infections are under-detected when co-occurring with Assemblage B. Additionally, Assemblage A may cause milder or more asymptomatic infections, meaning individuals are less likely to seek diagnosis. This could help explain why our study observed very few single A infections but a relatively high number of A and B mixed infections. Overall, the giardiasis infection rates peaked in spring and dropped in early autumn and winter. This is consistent with other reports of seasonality (Hoque et al. 2002, 2004). A peak incidence of giardiasis in NSW during October through to December coincides with high prevalence of outdoor and higher risk activities in these warmer months.

## Conclusions

In summary, this study provides new insights into the molecular diversity of *G. intestinalis* in NSW, Australia, and helps to inform enhanced surveillance and prevention strategies in developed metropolitan areas. During the study period, a higher prevalence of assemblage B was observed among human cases in NSW. Factors which possibly influence this higher incidence in NSW may be behavioural, climatic, environmental or related to the virulence or assemblage of the parasite. Higher numbers of mixed assemblage infections were also identified, which is a novel finding for a developed country like Australia. The distribution of assemblages A and B remained relatively uniform across genders and no clear differences were observed in clinical presentation between assemblages; however, assemblage B was more commonly observed among children. Further high-powered studies are needed to investigate the prevalence and clinical manifestations of assemblage B in children. While most giardiasis cases were transmitted locally, those individuals who had reported travelling overseas prior to illness onset were 6 times more likely to be infected with mixed assemblages A and B as opposed to single assemblages. This novel discovery underscores the importance of additional investigation into ‘travel’ as a risk factor for Australians, particularly delving into the differences observed in giardiasis transmission dynamics between endemic and international cases. Among metropolitan LHDs, *G. intestinalis* cases were consistently identified in the Nepean Blue Mountains, Northern Sydney, Western Sydney, South-eastern Sydney, Sydney CBD and Central Coast regions, which persisted throughout all seasons, and have highlighted these locations as potential disease hotspots in NSW. It remains essential to improve our knowledge of giardiasis and its molecular epidemiology among host populations; to help better inform surveillance strategies and response actions aimed at preventing further spread of infection. Further studies involving the geospatial and spatiotemporal distribution of *G. intestinalis* assemblages are recommended, and in particular targeting the metropolitan and urban areas of NSW.

## Supporting information

10.1017/S0031182025100991.sm001 Zajaczkowski et al. supplementary material 1 Zajaczkowski et al. supplementary material

10.1017/S0031182025100991.sm002 Zajaczkowski et al. supplementary material 2 Zajaczkowski et al. supplementary material

## Data Availability

The data that support the findings of this study are available from the corresponding author, [author initials], upon reasonable request.

## Supplementary material

The supplementary material for this article can be found at https://doi.org/10.1017/S0031182025100991.

## References

[ref1] Adam RD, Dahlstrom EW, Martens CA, Bruno DP, Barbian KD, Ricklefs SM, Hernandez MM, Narla NP, Patel RB, Porcella SF and Nash TE (2013) Genome Sequencing of *Giardia lamblia* Genotypes A2 and B Isolates (DH and GS) and Comparative Analysis with the Genomes of Genotypes A1 and E (WB and Pig). *Genome Biology and Evolution* 5, 2498–2511. 24307482 10.1093/gbe/evt197 PMC3879983

[ref2] Al-Mohammed HI (2011) Genotypes of *Giardia intestinalis* clinical isolates of gastrointestinal symptomatic and asymptomatic Saudi children. *Parasitology Research* 108, 1375–1381. 20838811 10.1007/s00436-010-2033-5

[ref3] Ankarklev J, Hestvik E, Lebbad M, Lindh J, Kaddu-Mulindwa DH, Andersson JO, Tylleskär T, Tumwine JK and Svärd SG (2012) Common coinfections of *Giardia intestinalis* and *Helicobacter pylori* in non-symptomatic Ugandan children. *PLoS Neglected Tropical Diseases* 6, e1780–e1780. 10.1371/journal.pntd.0001780. 22953010 PMC3429385

[ref4] Asher AJ, Holt DC, Andrews RM and Power ML (2014) Distribution of *Giardia duodenalis* Assemblages A and B among Children Living in a Remote Indigenous Community of the Northern Territory, Australia. *PLoS One Public Library of Science* 9, e112058. 10.1371/journal.pone.0112058 PMC4239041 25412502

[ref5] Asher AJ, Hose G and Power ML (2016) Giardiasis in NSW: Identification of *Giardia duodenalis* assemblages contributing to human and cattle cases, and an epidemiological assessment of sporadic human giardiasis. *Infection, Genetics and Evolution* 44, 157–161. 10.1016/j.meegid.2016.06.051 27370572

[ref6] Australian Bureau of Statistics (2017) *2016 Census - Demographics & Education*, 1030 (SUA).

[ref7] Bahrami F, Zamini GH, Haghighi A and Khademerfan MB (2017) Detection and molecular identification of human *Giardia* isolates in the West of Iran. *Biomedical Research (0970-938X)*; **28** Published online: 2017.

[ref8] Becker-Dreps S, Bucardo F, Vilchez S, Zambrana LE, Liu L, Weber DJ, Peña R, Barclay L, Vinjé J, Hudgens MG, Nordgren J, Svensson L, Morgan DR, Espinoza F and Paniagua M (2014) Etiology of Childhood Diarrhea Following Rotavirus Vaccine Introduction: A Prospective, Population-Based Study in Nicaragua. *The Pediatric Infectious Disease Journal* 33, 1156–1163. 10.1097/INF.0000000000000427 24879131 PMC4216626

[ref9] Breathnach AS, McHugh TD and Butcher PD (2010) Prevalence and clinical correlations of genetic subtypes of *Giardia lamblia* in an urban setting. *Epidemiology & Infection* 138, 1459–1467. 20144251 10.1017/S0950268810000208

[ref10] Coelho CH and Singer SM (2018) Recent advances in the *Giardia*-host relationship reveal danger lurking behind the smile. *PLoS Neglected Tropical Diseases* 12, e0006625. 30188894 10.1371/journal.pntd.0006625 PMC6126833

[ref11] Communicable Diseases Branch (2017) *Giardiasis notifications in NSW residents, by five year age group and gender. NSW Ministry of Health*. (https://www.health.nsw.gov.au/Infectious/Pages/data.aspx). Accessed 20 December 2021.

[ref12] Communicable Diseases Branch (2018) Health Protection NSW year in review 2017: Standard rate of disease per 100,000 population by local health district (LHD). Health Protection Report NSW.

[ref13] Couturier BA, Jensen R, Arias N, Heffron M, Gubler E, Case K, Gowans J and Couturier MR (2015) Clinical and analytical evaluation of a single-vial stool collection device with formalin-free fixative for improved processing and comprehensive detection of gastrointestinal parasites. *Journal of Clinical Microbiology* 53, 2539–2548. 10.1128/JCM.00838-15 26019199 PMC4508439

[ref14] Craun GF, Brunkard JM, Yoder JS, Roberts VA, Carpenter J, Wade T, Calderon RL, Roberts JM, Beach MJ and Roy SL (2010) Causes of outbreaks associated with drinking water in the United States from 1971 to 2006. *Clinical Microbiology Reviews* 23, 507–528. 10.1128/CMR.00077-09 20610821 PMC2901654

[ref15] Department of Education, Employment and Workplace Relations (2022) Child Care in Australia report Financial year 2018-19. *Child Care Package. Australian Government*.

[ref16] Dreelin EA, Ives RL, Molloy S and Rose JB (2014) *Cryptosporidium* and *Giardia* in surface water: A case study from Michigan, USA to inform management of rural water systems. *International Journal of Environmental Research and Public Health* 11, 10480–10503. 25317981 10.3390/ijerph111010480 PMC4210991

[ref17] Ebner J, Koehler AV, Robertson G, Bradbury RS, Jex AR, Haydon SR, Stevens MA, Norton R, Joachim A and Gasser RB (2015) Genetic analysis of *Giardia* and *Cryptosporidium* from people in Northern Australia using PCR-based tools. *Infection, Genetics and Evolution* 36, 389–395. 10.1016/j.meegid.2015.08.034 26321301

[ref18] Efstratiou A, Ongerth JE and Karanis P (2017) Waterborne transmission of protozoan parasites: Review of worldwide outbreaks - An update 2011–2016. *Water Research* 114, 14–22. 10.1016/j.watres.2017.01.036 28214721

[ref19] El Basha NR, Zaki MM, Hassanin OM, Rehan MK and Omran D (2016) *Giardia* Assemblages A and B in Diarrheic Patients: A Comparative Study in Egyptian Children and Adults. *Journal of Parasitology* 102, 69–74. 26509291 10.1645/14-676

[ref20] ElBakri A, Samie A, Bessong P, Potgieter N and Odeh RE (2014) *Detection and Molecular Characterisation of Giardia Lamblia Genotypes in Sharjah, United Arab Emirates. Transactions of the Royal Society of Tropical Medicine and Hygiene* 108, 466–473. 24906796 10.1093/trstmh/tru083

[ref21] Elhadad H, Abdo S, Tolba M, Salem AI, Mohamed MA, El-Abd EA and El-Taweel HA (2021) Detection of *Giardia intestinalis* assemblages A and B among children from three villages in the West Delta region, Egypt using assemblage specific primers. *Journal of Parasitic Diseases* 45, 655–663. 34475646 10.1007/s12639-020-01338-x PMC8368831

[ref22] Espelage W, an der Heiden M, Stark K and Alpers K (2010) Characteristics and risk factors for symptomatic *Giardia lamblia* infections in Germany. *BMC Public Health* 10, 41. 20105338 10.1186/1471-2458-10-41 PMC2824735

[ref23] Faria CP, Zanini GM, Dias GS, da Silva S and Sousa MD (2016) Molecular Characterization of *Giardia lamblia*: First Report of Assemblage B in Human Isolates from Rio de Janeiro (Brazil). *PLoS One Public Library of Science* 11, e0160762. 10.1371/journal.pone.0160762 PMC4982690 27517469

[ref24] Feng Y and Xiao L (2011) Zoonotic Potential and Molecular Epidemiology of *Giardia* Species and Giardiasis. *Clinical Microbiology Reviews* 24, 110–140. 21233509 10.1128/CMR.00033-10 PMC3021202

[ref25] Fletcher S, Caprarelli G, Merif J, Andresen D, Van Hal S, Stark D and Ellis JT (2014) Epidemiology and Geographical Distribution of Enteric Protozoan Infections in Sydney, Australia. *Journal of Public Health Research* 3, 298. 10.4081/jphr.2014.298 25343139 PMC4207027

[ref26] Fletcher S, Sibbritt D, Stark D, Harkness J, Rawlinson W, Andresen D, Van Hal S, Merif J and Ellis JT (2015) Descriptive epidemiology of infectious gastrointestinal illnesses in Sydney, Australia, 2007–2010. *Western Pacific Surveillance and Response Journal: WPSAR* 6, 7. 10.5365/WPSAR.2015.6.2.006 PMC4712528 26798556

[ref27] Geurden T, Geldhof P, Levecke B, Martens C, Berkvens D, Casaert S, Vercruysse J and Claerebout E (2008) Mixed *Giardia duodenalis* assemblage A and E infections in calves. *International Journal for Parasitology* 38, 259–264. 17854810 10.1016/j.ijpara.2007.07.016

[ref28] Haque R, Roy S, Kabir M, Stroup SE, Mondal D and Houpt ER (2005) *Giardia* Assemblage A Infection and Diarrhea in Bangladesh. *The Journal of Infectious Diseases* 192, 2171–2173. 16288384 10.1086/498169

[ref29] Helmy MM, Abdel-Fattah HS and Rashed L (2009) Real-time PCR/RFLP assay to detect *Giardia intestinalis* genotypes in human isolates with diarrhea in Egypt. *Journal of Parasitology* 95, 1000–1004. 19254068 10.1645/GE-1670.1

[ref30] Homan WL and Mank TG (2001) Human giardiasis: Genotype linked differences in clinical symptomatology. *International Journal for Parasitology* 31, 822–826. 10.1016/s0020-7519(01)00183-7 11403774

[ref31] Hopkins RM, Meloni BP, Groth DM, Wetherall JD, Reynoldson JA and Thompson RA (1997) Ribosomal RNA sequencing reveals differences between the genotypes of *Giardia* isolates recovered from humans and dogs living in the same locality. *The Journal of Parasitology* 83, 44–51. 9057695

[ref32] Hoque ME, Hope VT and Scragg R (2002) *Giardia* infection in Auckland and New Zealand: Trends and international comparison. *New Zealand Medical Journal* 115, 121. 12013302

[ref33] Hoque ME, Hope VT, Scragg R, Baker M and Shrestha R (2004) A descriptive epidemiology of giardiasis in New Zealand and gaps in surveillance data. *New Zealand Medical Journal* 117(1205), U1149. 15570332

[ref34] Huey CS, Mahdy MA, Al-Mekhlafi HM, Nasr NA, Lim YA, Mahmud R and Surin J (2013) Multilocus genotyping of *Giardia duodenalis* in Malaysia. *Infection, Genetics and Evolution* 17, 269–276. 10.1016/j.meegid.2013.04.013 23624189

[ref35] Hussein EM, Ismail OA, Mokhtar AB, Mohamed SE and Saad RM (2017) Nested PCR targeting intergenic spacer (IGS) in genotyping of *Giardia duodenalis* isolated from symptomatic and asymptomatic infected Egyptian school children. *Parasitology Research* 116, 763–771. 27975120 10.1007/s00436-016-5347-0

[ref36] Kashinahanji M, Haghighi A, Bahrami F, Fallah M, Saidijam M, Matini M and Maghsood AH (2019) *Giardia lamblia* assemblages A and B isolated from symptomatic and asymptomatic persons in Hamadan, west of Iran. *Journal of Parasitic Diseases* 43, 616–623. 31749533 10.1007/s12639-019-01139-x PMC6841826

[ref37] Kohli A, Bushen OY, Pinkerton RC, Houpt E, Newman RD, Sears CL, Lima AA and Guerrant RL (2008) *Giardia Duodenalis Assemblage, Clinical Presentation and Markers of Intestinal Inflammation in Brazilian Children. Transactions of the Royal Society of Tropical Medicine and Hygiene* 102, 718–725. 18485429 10.1016/j.trstmh.2008.03.002 PMC2963065

[ref38] Larkin MA, Blackshields G, Brown NP, Chenna R, McGettigan PA, McWilliam H, Valentin F, Wallace IM, Wilm A, Lopez R, Thompson JD, Gibson TJ and Higgins DG (2007) Clustal W and Clustal X version 2.0. *Bioinformatics* 23, 2947–2948. 10.1093/bioinformatics/btm404 17846036

[ref39] Lasek-Nesselquist E, Welch DM, Thompson RC, Steuart RF and Sogin ML (2009) Genetic exchange within and between assemblages of *Giardia duodenalis*. *Journal of Eukaryotic Microbiology* 57, 94. 10.1111/j.1550-7408.2009.00443.x 19883439

[ref40] Lebbad M, Petersson I, Karlsson L, Botero-Kleiven S, Andersson JO, Svenungsson B and Svärd SG (2011) Multilocus genotyping of human *Giardia* isolates suggests limited zoonotic transmission and association between assemblage B and flatulence in children. *PLoS Neglected Tropical Diseases* Public Library of Science 5, e1262–e1262. 10.1371/journal.pntd.0001262. 21829745 PMC3149019

[ref41] Lee J-H, Lee J, Park SJ, Yong TS and Hwang UW. (2006) Detection and genotyping of *Giardia intestinalis* isolates using intergenic spacers (IGS)-based PCR. *The Korean Journal of Parasitology* 44, 343–353. 17170576 10.3347/kjp.2006.44.4.343 PMC2559131

[ref42] Levecke B, Geldhof P, Claerebout E, Dorny P, Vercammen F, Cacciò SM, Vercruysse J and Geurden T (2009) Molecular characterisation of *Giardia duodenalis* in captive non-human primates reveals mixed assemblage A and B infections and novel polymorphisms. *International Journal for Parasitology* 39, 1595–1601. 19523472 10.1016/j.ijpara.2009.05.013

[ref43] Liu A, Zhang X, Zhang L, Wang R, Li X, Shu J, Zhang X, Shen Y, Zhang W and Ling H. (2012) Occurrence of bovine giardiasis and endemic genetic characterization of *Giardia duodenalis* *Isolates in Heilongjiang Province, in the Northeast of China. Parasitology Research* 111, 655–661. 22398834 10.1007/s00436-012-2883-0

[ref44] Mazumdar S, Fletcher-Lartey SM, Zajaczkowski P and Jalaludin B (2020) Giardiasis notifications are associated with socioeconomic status in Sydney, Australia: A spatial analysis. *Australian and New Zealand Journal of Public Health* 44, 508–513. 10.1111/1753-6405.13019 33197099

[ref45] Minetti C, Lamden K, Durband C, Cheesbrough J, Fox A, Wastling JM. (2015) Determination of *Giardia duodenalis* assemblages and multi-locus genotypes in patients with sporadic giardiasis from England. *Parasites & Vectors* 8, 444. 26338670 10.1186/s13071-015-1059-z PMC4559006

[ref46] Muhsen K and Levine MM (2012) A systematic review and meta-analysis of the association between *Giardia lamblia* and endemic pediatric diarrhea in developing countries. *Clinical Infectious Diseases* 55(Suppl 4), S271–293. 23169940 10.1093/cid/cis762 PMC3502312

[ref47] Mukherjee AK (2014) Association between *Giardia duodenalis* and Coinfection with Other Diarrhea-Causing Pathogens in India. *BioMed Research International* 2014, 786480. 10.1155/2014/786480. 25009820 PMC4070398

[ref48] Nolan MJ, Jex AR, Koehler AV, Haydon SR, Stevens MA and Gasser RB (2013) Molecular-based investigation of *Cryptosporidium* and *Giardia* from animals in water catchments in southeastern Australia. *Water Research* 47, 1726–1740. 23357792 10.1016/j.watres.2012.12.027

[ref49] Oliveira-Arbex AP, David EB, Oliveira-Sequeira TC, Bittencourt GN and Guimaraes ST (2016) Genotyping of *Giardia duodenalis* isolates in asymptomatic children attending daycare centre: Evidence of high risk for anthroponotic transmission. *Epidemiology & Infection* 144, 1418–1428. 26593069 10.1017/S0950268815002514 PMC9150566

[ref50] Painter JE, Gargano JW, Collier SA and Yoder JS (2015) Giardiasis Surveillance — United States, 2011–2012. *Morbidity and Mortality Weekly Report: Surveillance Summaries* 64, 15–25. 20535095

[ref51] Peréz Cordón G, Cordova Paz Soldan O, Vargas Vásquez F, Velasco Soto JR, Sempere Bordes LL, Sánchez Moreno M and Rosales MJ (2008) Prevalence of enteroparasites and genotyping of *Giardia lamblia* in Peruvian children. *Parasitology Research* 103, 459–465. 18470699 10.1007/s00436-008-1007-3

[ref52] Pestehchian N, Rasekh H, Babaei Z, Yousefi HA, Eskandarian AA, Kazemi M and Akbari M (2012) Identification of genotypes of *Giardia duodenalis* human isolates in Isfahan, Iran, using polymerase chain reaction–restriction fragment length polymorphism. *Advanced biomedical research* Wolters Kluwer–Medknow Publications; **1** Published online: 2012. 10.4103/2277-9175.105166 PMC3724326 23946932

[ref53] Plutzer J, Ongerth J and Karanis P (2010) *Giardia* taxonomy, phylogeny and epidemiology: Facts and open questions. *International Journal of Hygiene and Environmental Health* 213, 321–333. 20619729 10.1016/j.ijheh.2010.06.005

[ref54] Rafiei A, Roointan ES, Samarbafzadeh AR, Shayesteh AA, Shamsizadeh A, Borujeni MP (2013) Investigation of Possible Correlation between *Giardia duodenalis* Genotypes and Clinical Symptoms in Southwest of Iran. *Iranian Journal of Parasitology* 8, 389–395. 24454431 PMC3887239

[ref55] Rafiei A, Baghlaninezhad R, Köster PC, Bailo B, Hernández de Mingo M, Carmena D, Panabad E, Beiromvand M (2020) Multilocus genotyping of *Giardia duodenalis* in Southwestern Iran. A community survey. *PLoS One Public Library of Science* 15, e0228317. 10.1371/journal.pone.0228317 PMC7004373 32027684

[ref56] Read C, Walters J, Robertson ID, Thompson RC (2002) Correlation between genotype of *Giardia duodenalis* and diarrhoea. *International Journal for Parasitology* 32, 229–231. 11812501 10.1016/s0020-7519(01)00340-x

[ref57] Ryan U, Lawler S and Reid S (2017) Limiting swimming pool outbreaks of cryptosporidiosis - the roles of regulations, staff, patrons and research. *Journal of Water and Health* 15, 1–16. 10.2166/wh.2016.160 28151435

[ref58] Samie A, Tanih NF, Seisa I, Seheri M, Mphahlele J, ElBakri A, Mbati P (2020) Prevalence and genetic characterization of *Giardia lamblia* in relation to diarrhea in Limpopo and Gauteng provinces, South Africa. *Parasite Epidemiology and Control* 9, e00140. 32083192 10.1016/j.parepi.2020.e00140 PMC7016452

[ref59] Sarkari B, Ashrafmansori A, Hatam GR, Motazedian MH, Asgari Q, Mohammadpour I (2012) Genotyping of *Giardia lamblia* isolates from human in southern Iran. *Tropical Biomedicine* 29, 366–371. 23018499

[ref60] Savioli L, Smith H, Thompson A (2006) *Giardia* and *Cryptosporidium* join the ‘Neglected Diseases Initiative.’ *Trends in Parasitology* 22, 203–208. 16545611 10.1016/j.pt.2006.02.015

[ref61] Stark D, Al-Qassab SE, Barratt JL, Stanley K, Roberts T, Marriott D, Harkness J, Ellis JT (2011) Evaluation of multiplex tandem real-time PCR for detection of *Cryptosporidium* spp., *Dientamoeba fragilis, Entamoeba histolytica*, and *Giardia intestinalis* in clinical stool samples. *Journal of Clinical Microbiology* 49, 257–262. 21048004 10.1128/JCM.01796-10 PMC3020426

[ref62] Stucky BJ (2012) SeqTrace: A Graphical Tool for Rapidly Processing DNA Sequencing Chromatograms. *Journal of Biomolecular Techniques* 23, 90–93. 22942788 10.7171/jbt.12-2303-004 PMC3413935

[ref63] Sulaiman IM, Fayer R, Bern C, Gilman RH, Trout JM, Schantz PM, Das P, Lal AA, Xiao L (2003) Triosephosphate isomerase gene characterization and potential zoonotic transmission of *Giardia duodenalis*. *Emerging Infectious Diseases* 9, 1444–1452. 14718089 10.3201/eid0911.030084 PMC3035538

[ref64] Thompson RCA (2000) Giardiasis as a re-emerging infectious disease and its zoonotic potential. *International Journal for Parasitology* 30, 1259–1267. 10.1016/S0020-7519(00)00127-2 11113253

[ref65] Thompson RCA (2008) Giardiasis: Modern Concepts in Control and Management. *Annales Nestlé (English Ed.)* 66, 23–29. 10.1159/000113306

[ref66] Tungtrongchitr A, Sookrung N, Indrawattana N, Kwangsi S, Ongrotchanakun J, Chaicumpa W (2010) *Giardia intestinalis* in Thailand: Identification of Genotypes. *Journal of Health, Population, and Nutrition* 28, 42–52. 20214085 10.3329/jhpn.v28i1.4522 PMC2975845

[ref67] Waldron LS, Ferrari BC, Cheung-Kwok-Sang C, Beggs PJ, Stephens N and Power ML (2011) Molecular epidemiology and spatial distribution of a waterborne cryptosporidiosis outbreak in Australia. *Applied and Environmental Microbiology* 77, 7766–7771. 10.1128/AEM.00616-11 21908623 PMC3209151

[ref68] Wang Y, Gonzalez-Moreno O, Roellig DM, Oliver L, Huguet J, Guo Y, Feng Y, Xiao L (2019) Epidemiological distribution of genotypes of *Giardia duodenalis* in humans in Spain. *Parasites & Vectors* 12, 1–10. 31492183 10.1186/s13071-019-3692-4 PMC6728964

[ref69] Weitzel T, Dittrich S, Möhl I, Adusu E, Jelinek T (2006) Evaluation of seven commercial antigen detection tests for *Giardia* and *Cryptosporidium* in stool samples. *Clinical Microbiology and Infection* 12, 656–659. 16774562 10.1111/j.1469-0691.2006.01457.x

[ref70] Woschke A, Faber M, Stark K, Holtfreter M, Mockenhaupt F, Richter J, Regnath T, Sobottka I, Reiter-Owona I, Diefenbach A, Gosten-Heinrich P (2021) Suitability of current typing procedures to identify epidemiologically linked human *Giardia duodenalis* isolates. *PLoS Neglected Tropical Diseases* 15, e0009277. 33764999 10.1371/journal.pntd.0009277 PMC8023459

[ref71] Xiao L and Feng Y (2017) Molecular epidemiologic tools for waterborne pathogens *Cryptosporidium* spp. and *Giardia duodenalis*. *Food and Waterborne Parasitology* 8, 14–32. 32095639 10.1016/j.fawpar.2017.09.002 PMC7034008

[ref72] Yang R, Lee J, Ng J, Ryan U (2010) High prevalence *Giardia duodenalis* assemblage B and potentially zoonotic subtypes in sporadic human cases in Western Australia. *International Journal for Parasitology* 40, 293–297. 19703458 10.1016/j.ijpara.2009.08.003

[ref73] Yoder JS, Gargano JW, Wallace RM and Beach MJ (2012) Giardiasis Surveillance — United States, 2009–2010. *Morbidity and Mortality Weekly Report: Surveillance Summaries* 61, 13–23. 22951494

[ref74] Zahedi A, Field D and Ryan U (2017) Molecular typing of *Giardia duodenalis* in humans in Queensland – First report of Assemblage E. *Parasitology* 144, 1154–1161. 28482937 10.1017/S0031182017000439

[ref75] Zajaczkowski P, Lee R, Fletcher-Lartey SM, Alexander K, Mahimbo A, Stark D and Ellis JT (2021) The controversies surrounding Giardia intestinalis assemblages A and B. *Current Research in Parasitology & Vector-Borne Diseases* 1, 100055. 10.1016/j.crpvbd.2021.100055 35284870 PMC8906113

[ref76] Zajaczkowski P, Mazumdar S, Conaty S, Ellis JT and Fletcher-Lartey SM (2018) Epidemiology and associated risk factors of giardiasis in a peri-urban setting in New South Wales Australia. *Epidemiology and Infection* 147, e15. 10.1017/S0950268818002637 30264685 PMC6520257

