# Supplementary material for: Genotyping and distribution of Giardia intestinalis assemblages in NSW, Australia
Source: Parasitology. 2025 Oct 24;153(1):43–55. doi: 10.1017/S0031182025100991 (PMC13215750; doi:10.1017/S0031182025100991)
Supplement: Zajaczkowski et al. supplementary material 1 — Zajaczkowski et al. supplementary material [file S0031182025100991sup001.pdf]

Supplementary Table 1. Summary of sample numbers included in each group analysis and genotyping outcome.

Category	Samples (*n*)	%	Details
Total samples selected for genotyping	169	100%	Subset of full cohort with sufficient DNA for molecular analysis
**Genotyping Outcomes**			
Amplified at ≥ locus	162/169	95.9%	Assemblage A: 9.3% (15); B: 46.9% (76); A + B: 43.8% (71)
Successfully typed at *tpi* locus	147/169	87.0%	Assemblage A: 18.4% (27); B: 54.4% (80); A + B: 27.2% (40)
Successfully typed at SSU *rRNA* locus	136/169	80.5%	Assemblage A: 18.4% (25); B: 73.5% (100); A + B: 8.1% (11)
**Metadata Availability**			
Age data	145/169	85.8%	Cases with valid age information
Gender data	154/169	91.1%	Cases with valid gender information
Age and Gender data combined	145/169	85.8%	Cases with both age and gender data
Travel history data	129/169	76.3%	Includes any reported travel history
Clinical data	129/169	76.3%	Includes symptom and risk factor information
Geographic location data	141/169	83.4%	Based on submitting laboratory location
Seasonality data	140/169	82.8%	Includes specimen collection season
