# Supplementary material for: Genotyping and distribution of Giardia intestinalis assemblages in NSW, Australia
Source: Parasitology. 2025 Oct 24;153(1):43–55. doi: 10.1017/S0031182025100991 (PMC13215750; doi:10.1017/S0031182025100991)
Supplement: Zajaczkowski et al. supplementary material 2 — Zajaczkowski et al. supplementary material [file S0031182025100991sup002.pdf]

Supplementary Table 2. Co-infecting pathogens identified in *G. intestinalis* positive faecal samples

	Assemblage type, % (*n*)			
**Co-infecting pathogen(s)[a]**	A	B	A+B	**Total, % (*n*)**
Adenovirus* & *Dientamoeba fragilis	0.0% (0)	5.0% (2)	0.0% (0)	**2.5% (2)**
Adenovirus* & *Enterovirus	0.0% (0)	2.5% (1)	0.0% (0)	**1.3% (1)**
Adenovirus*, *Blastocystis hominis* & *Dientamoeba fragilis	0.0% (0)	2.5% (1)	0.0% (0)	**1.3% (1)**
Astrovirus* & *Blastocystis hominis	0.0% (0)	0.0% (0)	3.2% (1)	**1.3% (1)**
Astrovirus* & *Sapovirus	0.0% (0)	2.5% (1)	0.0% (0)	**1.3% (1)**
***Astrovirus*, *Blastocystis hominis*, *Sapovirus* & *Shigella* spp.**	0.0% (0)	2.5% (1)	0.0% (0)	**1.3% (1)**
Blastocystis hominis	22.2% (2)	32.5% (13)	32.3% (10)	**31.3% (25)**
Blastocystis hominis* & *Bocavirus	0.0% (0)	2.5% (1)	0.0% (0)	**1.3% (1)**
***Blastocystis hominis* & *Campylobacter* spp.**	0.0% (0)	0.0% (0)	3.2% (1)	**1.3% (1)**
Blastocystis hominis* & *Dientamoeba fragilis	11.1% (1)	10.0% (4)	9.7% (3)	**10.0% (8)**
Blastocystis hominis* & *Enterovirus	0.0% (0)	2.5% (1)	0.0% (0)	**1.3% (1)**
***Blastocystis hominis* & *Shigella* spp.**	0.0% (0)	0.0% (0)	3.2% (1)	**1.3% (1)**
Blastocystis hominis*, *Dientamoeba fragilis*, *Enterovirus* & *Sapovirus	0.0% (0)	2.5% (1)	0.0% (0)	**1.3% (1)**
***Campylobacter* spp.**	11.1% (1)	0.0% (0)	9.7% (3)	**5.0% (4)**
***Campylobacter* spp., *Enterovirus* & *Shigella* spp.**	0.0% (0)	2.5% (1)	3.2% (1)	**2.5% (2)**
Clostridium difficile	0.0% (0)	7.5% (3)	0.0% (0)	**3.8% (3)**
***Cryptosporidium* spp.**	11.1% (*n* = 1)	0.0% (*n* = 0)	3.2% (*n* = 1)	**2.5% (*n* = 2)**
Dientamoeba fragilis	22.2% (*n* = 2)	12.5% (*n* = 5)	16.1% (*n* = 5)	**15.0% (*n* = 12)**

Dientamoeba fragilis* & *Enterovirus	11.1% (*n* = 1)	0.0% (*n* = 0)	0.0% (*n* = 0)	**1.3% (*n* = 1)**
***Dientamoeba fragilis* & *Norovirus* GII**	0.0% (*n* = 0)	2.5% (*n* = 1)	0.0% (*n* = 0)	**1.3% (*n* = 1)**
Enterovirus	11.1% (*n* = 1)	5.0% (*n* = 2)	3.2% (*n* = 1)	**5.0% (*n* = 4)**
***Norovirus* GII**	0.0% (*n* = 0)	2.5% (*n* = 1)	0.0% (*n* = 0)	**1.3% (*n* = 1)**
***Salmonella* spp.**	0.0% (*n* = 0)	0.0% (*n* = 0)	3.2% (*n* = 1)	**1.3% (*n* = 1)**
Sapovirus	0.0% (*n* = 0)	0.0% (*n* = 0)	6.5% (*n* = 2)	**2.5% (*n* = 2)**
Yersinia enterocolitica	0.0% (*n* = 0)	2.5% (*n* = 1)	3.2% (*n* = 1)	**2.5% (*n* = 2)**
**Total**	**100.0% (*n* = 9)**	**100.0% (*n* = 40)**	**100.0% (*n* = 31)**	**100.0% (*n* = 80)**

[a]As reported by the real-time PCR (RT-PCR) EasyScreen™ assay
